# Specific and Individuated Death Reflection Fosters Identity Integration

**DOI:** 10.1371/journal.pone.0154873

**Published:** 2016-05-06

**Authors:** Laura E. R. Blackie, Philip J. Cozzolino, Constantine Sedikides

**Affiliations:** 1 Department of French and Francophone Studies, University of Nottingham, Nottingham, Nottinghamshire, United Kingdom; 2 Department of Psychology, University of Essex, Colchester, Essex, United Kingdom; 3 School of Psychology, University of Southampton, Southampton, Hampshire, United Kingdom; Virginia Commonwealth University, UNITED STATES

## Abstract

Identity integration is the process wherein a person assimilates multiple or conflicting identities (e.g., beliefs, values, needs) into a coherent, unified self-concept. Three experiments examined whether contemplating mortality in a specific and individuated manner (i.e., via the death reflection manipulation) facilitated outcomes indicative of identity integration. Participants in the death reflection condition (vs. control conditions) considered positive and negative life experiences as equally important in shaping their current identity (Experiment 1), regarded self-serving values and other-serving values as equally important life principles (Experiment 2), and were equally motivated to pursue growth-oriented and security-oriented needs (Experiment 3). Death reflection motivates individuals to integrate conflicting aspects of their identity into a coherent self-concept. Given that identity integration is associated with higher well-being, the findings have implications for understanding the psychological benefits of existential contemplation.

## Introduction

For a meaning-making species, acknowledging mortality’s inevitability can offer a unique lens through which to view the self and its place in the world. This theme echoes in many literary and cinematic works. A classic example is Charles Dickens’s character Ebenezer Scrooge, who amends his selfish and uncompassionate ways after he realizes how little others care about his eventual demise. Pondering their mortality may offer people an opportunity to take stock of their life experiences, reconsider their values or needs, and re-configure their identity in tune with the newly acquired insights. We address these possibilities in the current article. We test whether death reflection influences (a) the importance that individuals place on their past experiences, values, or needs, and (b) the integration of these experiences, values, or needs into a coherent self-concept.

### Mortality Reminders and the Self-Narrative

Reflecting on one’s mortality intensifies the search for meaning in life [1]. But how does such a search lead to re-construction of one’s self-narrative as a lucid, coherent account? A coherent self-narrative can buffer against fear of death, because the self-narrative weaves together seemingly fleeting or inconsequential aspects of one’s life into a more meaningful story that connects the individual to a broader and enduring cultural system [2]. Consistent with this proposition, participants who value structure and organization in their life construct a coherent, clearly-defined, and simply organized self-narrative following a mortality reminder. Also, after a mortality cue, participants emphasize in their self-narratives the significance of connections among their past, present, and future [3, 4, 5].

Relatedly, anecdotal evidence from the near-death experience literature suggests that individuals can sometimes undergo considerable change in their self-perception and outlook on life [6] Those who have had a near-death experience often report higher self-worth, more compassion, greater appreciation of life, decreased fear of death, and reorganization of priorities with ascription of diminished importance to materialistic values [7, 8, 9]. The life review process is considered the key component of the near-death experience and responsible for these after-effects [6]. During life review, individuals are said to re-live some of the most pivotal events of their life over again. Some persons claim that this re-living process enables them to understand themselves more clearly and to appreciate the interconnectedness of their life to others’ and to the natural world. Thus, the near-death experience, as aversive as it may be, can motivate some persons to engage in an assessment of their lives, and to reconsider their beliefs, values, or needs.

### Identity Integration and Well-Being

Although the influence of mortality cues on the organization of self-narratives has been well-documented [2, 3], far less empirical attention has been directed at whether individuals integrate different, and at times conflicting, identities (e.g., beliefs, values, needs) following a mortality reminder. For example, individuals may hold values that appear incompatible at first glance, such as conforming to social expectations and pursuing non-traditional paths [10]. Yet, both values are integral components of the self-concept and, as such, will need to be reconciled. The process of assimilating divergent identities into a coherent self-concept is known as identity integration [11, 12]. Stated otherwise, identity integration refers to individuals internalizing the importance of a particular identity and bringing it into harmony with existing ones [13, 14]. In a similar vein, philosophical and psychological theories of wisdom have proposed that identity integration, a balanced and coordinated pursuit of both self-oriented interests and other-oriented interests, is at the core of a good life and optimal well-being [15].

In this article, we examine whether a mortality awareness manipulation—modeled on the near-death experience—motivates individuals to integrate conflicting identities into a coherent self-concept. We operationalize identity integration in terms of ascription of equal importance to two contradictory identities (again: beliefs, values, or needs) as opposed to downplaying the importance of one identity [14]. We concur with contemporary perspectives of wisdom, which posit that balancing conflicting identities is at the heart of healthy and optimal human development [15]. We are interested in how situationally-imposed mortality reminders impact upon identity integration, and we thus focus on state rather than trait or chronic cf. [16, 17] processes.

Identity integration is associated with greater levels of psychological adjustment, maturity, and well-being. Bicultural individuals who have integrated their dual-nationalities report higher well-being compared to those with fragmented nationality-related identities [18] [19]. Additionally, individuals who form self-narratives that feature integrative perspectives on the self, others, and the world report higher maturity, ego-development, and well-being [20, 21]. These individuals are better able to reconfigure their identities so as to derive meaning from mildly negative life events, and consequently report higher self-acceptance, satisfaction with life, and self-esteem, as well as lower depression [22]. In contrast, identity disintegration or a fragmented self-concept is associated with lower self-esteem and greater depression [23, 24, 25]. A severely fragmented self-concept may also be symptomatic of personality (e.g., psychotic) disorders [26].

### Facilitating Identity Integration via Mortality Awareness

We investigate the extent to which identity integration is facilitated by the manner in which participants construe their mortality. Following on from recent work that has outlined the more positive trajectories of mortality awareness [27, 28], we wondered about the psychological processes that may be responsible for triggering these less defensive or more constructive responses to thoughts of mortality. Individuals’ reaction to mortality can vary from attempts to distance themselves from its inevitability to efforts toward reconciling its certainty and integrating it into their self-concept [29, 30]. This notion is at the foundation of the dual-existential systems model [31], which proposes that individuals’ reaction to a mortality cue is influenced, at least in part, by the manner in which they construe death.

According to the dual-existential systems model, individuals are motivated to distance themselves from the reality of their mortality when they construe the relevant reminder in an abstract or decontextualized manner. Hence, they will engage in compensatory behavior to diminish the threat associated with the mortality reminder. The outcome of such behavior could be positive or negative. The literature has established that subtle mortality cues, such as subliminal primes of the word ‘dead’ or expression of one’s thoughts about dying, cause participants to uphold their beliefs and values [32]. Depending on the situation, this process can facilitate the derogation of others who challenge participants’ beliefs or can enable stronger expressions of positive beliefs and standards such as prosocial values [27]. These processes allow participants to affirm their cultural worldviews and, as a result, to feel part of a cultural system that is larger and more enduring than themselves [33, 34, 35].

In contrast, persons who have had a near-death experience often report more profound changes to their beliefs, values, and needs [7, 9]. Existential awareness, then, may sometimes goad individuals into re-configuring their identity. Indeed, experimental research has shown that under certain circumstances mortality manipulations can foster cultural exploration and depreciation of extrinsic values [27]. What are the circumstances, however, that trigger growth-oriented responses? We propose, based on the dual-existential systems model [31], that one such circumstance is construal of mortality in a specific and individuated manner. Construing mortality in this manner stirs individuals to engage with the significance of this inevitable reality, and, similar to the near-death experience, to take stock of their lives by reflecting on their beliefs, values, and needs. It is through this type of mortality construal that people become aware of what is important to them and motivated to integrate these identities into a coherent self-concept.

To test whether specific and individuated mortality awareness instigates growth-oriented responses, [36] designed the death reflection (DR) manipulation. Participants read a vivid, evocative, and detailed 300-word scenario in which they imagine themselves inescapably dying in an apartment fire. The scenario is followed by four open-ended questions. The first two questions invite participants to describe their thoughts and feelings about the event, and their beliefs about the way in which they would have handled their final moments. Of particular relevance are the last two questions. These are modeled on the life review process said to be integral to induction of the constructive after-effects of the near-death experience [6]. The questions invite participants to describe the life they would have led up to that point and how their family would react if this event were to happen to them.

Findings have been consistent with the dual-existential systems model. The DR participants most concerned with extrinsic rewards demonstrated less greed and reported feeling more spiritual compared to those subjects exposed to an established and widely-used mortality manipulation (i.e., mortality salience or MS) [37] and compared to those in scenario-based control conditions [36]. Also, DR participants’ express stronger intentions to donate blood—intentions unaffected by the magnitude of community needs for blood donation—compared to MS and control participants [38]. Finally, when the standard MS manipulation is modified to induce a limited-time perspective through instructions to imagine one’s death as a healthy 75-year-old, participants demonstrate lower greed and higher cooperation in a prisoner’s dilemma game [39]. In all, reactions to mortality reminders can differ depending on the manner in which individuals construe death.

### Current Research

In three experiments, we assess whether specific and individuated death construal (via DR) produces outcomes indicative of identity integration. In Experiment 1, we examine participants’ beliefs, focusing on whether DR participants are likely to accept both positive *and* negative life events as relevant to their past and present selves. This outcome is symptomatic of an integrative self-schema in which individuals incorporate both positive and negative identities rather than compartmentalizing them into separate self-schemas [40]. Next, we examine whether DR participants give equal weight to seemingly opposing values (Experiment 2) and needs (Experiment 3) rather than downplaying one (value or need, respectively) at the expense of the other. Our hypothesis stipulates that it is specific and individuated construal of death that facilitates identity integration rather than more general thoughts of death. For that reason, we compare the DR manipulation with two control conditions: the standard MS manipulation and a non-existential condition (i.e., a visit to the dentist).

## Experiment 1: Life Event Integration

In Experiment 1, we examine identity integration in the context of past events. Identity integration implies that individuals are capable of accepting the relevance of negative life events to shape their self-concept [14]. It is not surprising that individuals readily accept the relevance of positive life events, because positive memories are associated with wellness, comfort, and pleasant affect [41, 42, 43]. The ease with which individuals accept negative life events, however, is more nuanced [44, 45, 46]. For some, negative life experiences are simply something to be weathered or forgotten, but, for others, they present an opportunity for self-improvement and personal growth [47, 48]. These differences are paralleled at the structural level. People can organize their self-concept in an integrative manner and incorporate both positive and negative experiences, or in a compartmentalized manner and incorporate only positive or only negative life experiences [49, 50]. An integrative self-concept therefore presents a less biased picture of the self-concept, as people attribute equal weight to the importance of both positive and negative experiences when they gauge the relevance of these experiences in forming their current identity.

In Experiment 1, we examine whether specific and individuated death awareness (DR) increases the likelihood that participants will organize their self-concept in an integrative manner compared to those in both control conditions (i.e., the MS and dentist conditions). We tested this hypothesis by using a validated identity integration task [14] that instructs participants to recall both a positive and a negative life experience and then, afterwards, asks them to rate the extent to which each experience has shaped their current identity. We hypothesized that DR would facilitate identity integration, and, as such, participants would indicate that both positive and negative life experiences were equally important in shaping their identity. In contrast, and in line with [14] findings, participants in the control conditions (i.e., the MS and dentist conditions) would show a bias in favor of positive experiences, and, as such, they would consider positive (relative to negative) experiences more influential in shaping their identity.

### Method

#### Participants

We tested 108 student and staff volunteers from the University of Essex aged 18–70 years (*M* = 28.81, *SD* = 14.62). The majority of participants were White British (78.7%) or White European (13.9%) followed by Black Caribbean (1.9%) and Chinese (0.9%). 4.6% of participants did not specify their race. The core design was a 3 (condition: DR, MS, Dentist) X 2 (recall order: first, second) between-subjects factorial. The MS and dentist conditions were control conditions. We added event acceptance (positive, negative, neutral) as a repeated measures factor (see below). The Ethics Committee of the University of Essex approved the experiment. All the participants provided informed written consent prior to participation.

#### Materials and Methods

We randomly assigned participants to the DR condition or one of two control conditions. We have already described the DR manipulation in detail [36]. With regard to the two control conditions, participants completed either a standard MS manipulation in which they answered two open-ended questions about their thoughts on death and dying [37], or a non-existential condition in which they answered two parallel open-ended questions about a dental procedure. Afterwards, participants completed a word search puzzle that is used normatively as a distraction task in experimental existential research to remove death-related cognitions from focal attention [32]. Participants then responded to the dependent measure. They completed the event acceptance measure, a modified version of a validated identity integration task [14]. They recalled a positive, negative, and neutral event that they had experienced one year prior to the experiment. We counterbalanced the order of the positive and negative life events, with all participants recalling the neutral event last. We modified the task to include a neutral event condition, and we did not include an additional one-item dependent variable that asked whether participants recalled the event using a first- or third-person perspective. In all other respects, we presented the instructions and dependent variables exactly as in [14].

Specifically, we instructed participants to take a few moments to recall a life event that “had a strong impact on you, making you feel happy and contented” (positive event condition), “had a strong impact on you, making you feel shameful and regretful” (negative event condition), and “had no real impact on you, making you feel no different from usual” (neutral event condition). These instructions were followed by an empty text box in which participants described the event in as much detail as possible. After each recollection, participants responded to four items assessing the extent to which they accepted the event as part of their lives or as integral to who they are today (1 = not at all true, 9 = extremely true). The items were: “I accept the experience I had,” “I embrace that this event is part of my past,” “I feel that this event was an important part of my past,” and “I think that this event has helped to make me the person I am today” (positive event α = .65; negative event α = .70; neutral event α = .76). The original validation of this identity integration task by [14] demonstrated that the positive framing of the dependent variables did not encourage participants to respond in a socially desirable manner, as participants in one condition were significantly more defensive in their responses to the negative event condition. Furthermore, the anticipated integration effect remained significant when [14] controlled for trait self-esteem.

### Results and Discussion

We conducted a 3 (condition: DR, MS, dental pain) X 2 (recall order: first, second) X 3 (event acceptance: positive event, negative event, neutral event) mixed Analysis of Variance (ANOVA), with the third factor being repeated measures. Neither the recall order main effect nor any interactions involving this factor were significant. The main effects for condition, *F*(2, 102) = 5.46, *p* < .01, η_p_^2^ = 0.10, and event acceptance, *F*(2, 204) = 66.38, *p* < .01, η_p_^2^ = 0.39, were significant. However, they were qualified by the crucial Condition X Event Acceptance interaction, *F*(4, 204) = 2.67, *p* < .05, η_p_^2^ = 0.05 (Table 1).

**Table 1:** Means and Standard Deviations for Event Acceptance as a Function of Condition in Experiment 1.

Condition	Event Acceptance					
	Positive Event		Negative Event		Neutral Event	
	Mean	SD	Mean	SD	Mean	SD
DR	7.81	1.44	7.40	1.32	5.37	2.13
MS	7.15	1.10	5.61	1.78	4.96	1.95
Non-Existential	7.21	1.61	6.42	1.74	5.28	1.85

We broke down this interaction in a theoretically relevant way, that is, through three paired sampled t-tests. We compared, then, positive and negative event acceptance scores within each condition. Positive and negative events were equally important in shaping the current identity of DR participants, *t*(35) = 1.45, *p* = 0.16. In contrast, positive events had a stronger influence than negative events in shaping the current identity of both MS participants, *t*(35) = -5.80, *p* < 0.05, and non-existential control participants *t*(35) = -2.52, *p* < 0.05. Neutral events received the lowest ratings in all three conditions (Table 1). This is not surprising, as we did not expect events that made participants feel “no different from usual” to have an influential role in identity formation. Indeed, the influence of neutral events did not differ across conditions, *F*(2, 105) = 0.43, *p* = .65.

Taken together, participants who contemplated their death in a specific and individuated manner indicated that positive and negative experiences were equally impactful in shaping their current identity. In contrast, and consistent with [14], participants in the two control conditions indicated that positive (relative to negative) experiences were more impactful in shaping their identity. The findings support the assumption that accepting the role that negative life experiences have had in one’s life is a challenging process [44, 45, 46] and may only occur under certain conditions. Specific and individuated consideration of mortality constitutes such a condition facilitating identity integration.

## Experiment 2: Value Integration

In Experiment 2, we examined identity integration in the context of values. Identity integration is a dynamic process in which a person acknowledges all aspects of who they are and brings their values (as well as beliefs or needs) into harmony with one another. Values are cognitive representations of personally meaningful goals [51, 52] and serve as life principles that guide the interpretation of the self, others, and external events [53, 54]. Thus, similar to the integration of life experiences [14], values—even conflicting ones—are fully incorporated into an individual’s self-concept when they reflect fundamental aspects of their identity [11]. Additionally, as discussed earlier, contemporary theories of wisdom claim that a core component of the good life is the balanced pursuit of self-serving and other-serving values. For example, [15] have argued that “a wise person does not prefer and pursue self-serving values at the expense of other-serving values and vice versa” (p. 342). At the crux of identity integration, then, is the notion of balance: individuals recognize the importance of both types of values and therefore do not single-mindedly pursue one at the expense of the other.

Values are malleable to some extent. As the demands of a situation change, so does the relative importance that persons ascribe to certain values [55, 56]. Additionally, mortality primes influence the values that persons momentarily endorse. For example, after a MS manipulation, participants place greater importance on values that emphasize connections to their culture and similar-minded others [57, 58]. The explanation for this results pattern is that persons cope with the threat of death by feeling part of a larger and more enduring cultural system, a system that also validates their core beliefs or values about the world [33, 57, 59]. Yet, based on Experiment 1 findings, we hypothesized that specific and individuated reminders of mortality would facilitate the integration of conflicting values.

We tested this hypothesis in Experiment 2. In particular, we examined whether specific and individuated death contemplation (DR) results in the integration of conflicting values, namely, self-serving values and other-serving values. We focused on the openness to change and conservation dimensions of the Schwartz Value Inventory (SVI) [10]. These value dimensions capture the tension between the motivation to pursue one’s intellectual and emotional interests (openness to change values) on the one hand, and the motivation to preserve the social status quo and remain embedded in a larger social community (conservation values) on the other. As pivotal as these values are [10], it is difficult for persons to endorse them simultaneously [56].

We hypothesized that DR would facilitate identity integration, and, as such, participants would rate the two values as equally important. In contrast, we hypothesized that the two control conditions would show a bias in favor of one value dimension, but that this preference would differ between the two conditions. Based on the literature [57, 58], we hypothesized that MS participants would place greater importance on conservation values, as these values emphasize cultural connectedness. However, non-existential control participants would place greater importance on openness to change values, as the motivation to pursue one’s intellectual and emotional interests is likely to be potent among members of an independent culture (i.e., UK), given the cultural emphasis on values of individualism and uniqueness [60].

### Method

#### Participants

We tested 81 student and staff volunteers (56 women, 25 men) from the University of Essex aged 18–64 years (*M* = 31.32, *SD* = 13.05). The majority of participants were Arab (50.6%) or White British (38.3%) followed by White European (3.7%), Pakistani (3.7%), Bangladeshi (1.2%) and Indian (1.2%). 1.2% of participants did not specify their race. Furthermore, 58% of participants were not UK citizens. The design was a 3 (condition: DR, MS, non-existential) x 2 (value dimension: openness to change, conservation) mixed factorial, with condition as a between-subjects factor and value dimension as a within-subjects factor. The Ethics Committee of the University of Essex approved the experiment. All the participants provided informed written consent prior to participation.

#### Materials and Method

We randomly assigned participants to the three between-subjects conditions. After a filler task, they completed the 57-item SVI [10]: they rated each value on its importance as “a guiding principle” in their lives (-1 = opposed to my values, 7 = of supreme importance). Based on [10] instructions, we asked participants (a) to distinguish as much as possible between values by using all the numbers, and (b) that in most circumstances participants rate only two of the values on the list as a 7.

### Results and Discussion

We formed the openness to change dimension by averaging the stimulation and self-direction values (α = .63), and the conservation dimension by averaging the conformity, security, and tradition values (α = .86). We conducted a 3 (condition) X 2 (value dimension) ANOVA, with the second factor being repeated measures. We controlled for individual differences in the scale by including each participant’s averaged score across all items as a covariate, as is standard practice in use of the SVI (for the coding manual see [61]). The condition main effect was not significant, *F*(2, 77) = 0.33, *p* = .72, but the value dimension main effect was significant, *F*(1, 77) = 7.28, *p* < .01, η_p_^2^ = 0.09. The crucial Condition X Value Dimension interaction was significant, *F*(2, 77) = 4.97, *p* < .01, η_p_^2^ = 0.11 (Table 2).

**Table 2:** Means and Standard Deviations for Openness to Change and Conservation Values as a Function of Condition in Experiment 2.

Condition	Openness to Change Values		Conservation Values	
	Mean	SD	Mean	SD
DR	4.83	0.96	4.79	0.93
MS	4.21	1.03	4.70	0.80
Non-Existential	4.40	0.81	3.93	1.48

We broke down this interaction through three theoretically relevant paired sampled t-tests. In particular, we compared the openness to change and conservation scores within each condition. The two values were equally important guiding principles in life for DR participants, *t*(26) = 0.24, *p* = 0.81. Additionally, conservation values were more important guiding life principles than openness to change values for MS participants, *t*(26) = 2.64, *p* < 0.01. The non-existential control participants did not rate the openness values as more important guiding life principles than the conservative values, but this effect was marginal, *t*(26) = 1.79, *p* = 0.09.

In all, participants who contemplated their death in a specific and individuated manner demonstrated evidence of identity integration, given that they rated two conflicting value dimensions as equally important life guiding principles. In contrast, participants in the MS condition rated conservation values as more important than openness to change values. This is consistent with past findings that the MS manipulation increases participants’ desire to feel connected to a larger cultural system and to people with similar values [57, 33]. Inconsistent with our hypotheses, participants in the non-existential control condition did not rate openness values as more important guiding life principles than conservation values, although the effects were in the predicted direction (*p* = 0.09).

## Experiment 3: Need Integration

In Experiment 3, we examined identity integration in the context of needs. Individuals are motivated by two independent regulatory orientations: need for growth or accomplishment and need for security or safety [62]. The salience of these needs may differ chronically between individuals and can also be temporally induced by primes [63]. Ordinarily, people pursue a goal motivated by one of these regulatory orientations, even though the orientation may differ depending on the particular goal. However, in light of the results of Experiments 1–2, we examined whether specific and individuated mortality awareness (vs. controls) would influence the importance attributed to growth and security-oriented needs. As discussed earlier, identity integration is a dynamic process in which persons acknowledge all aspects of who they are and brings their beliefs, values, and needs into harmony with one another [11, 14]. Additionally, psychological needs may be theorized to contribute to the good life in the same way that the balanced and coordinated pursuit of values has been [15]. An individual cannot thrive in situations in which she single-mindedly pursues either security or advancement needs; rather, she must recognize the relevance of both.

In Experiment 3, we examined whether specific and individuated death awareness increases the likelihood that the two conflicting needs, the need for advancement and the need for security, will be integrated. For that purpose, we modified an experimental task developed by [64] that subtly activates the two needs. Participants complete a maze in which they guide a mouse either to nourishment (i.e., cheese) or safety (i.e., away from an owl). The mazes activate semantic and procedural representations associated with advancement needs such as approaching nourishment, or security needs such as avoiding danger [63]. The task has been implemented extensively in investigations on the influence of growth or security needs upon creativity, attention, and analytical problem-solving [65]. We modified the task for use as a dependent variable to test whether the relevance of growth and security needs differs as a function of specific and individuated mortality awareness. Participants undertook the DR manipulation (vs. controls) and then were asked to provide feedback on two computerized mazes. They learned that, based on their feedback, only one maze would be developed and applied in future research. After reading a description of each maze game, participants stated how motivated they would be to play each game.

We hypothesized that DR would facilitate identity integration. Participants in this condition would view the needs as equally important to their identity, and, as a consequence, would be equally motivated to play the maze games. However, participants in the control conditions (i.e., the MS or dentist condition) would lean in favor of one maze (i.e., the security-based maze), and, as such, would be more strongly motivation to play this maze. We reasoned that control participants would favor the security maze, because it is more representative of a typical computer game in which the player must pursue a goal (i.e., safety) while avoiding obstacles (i.e., a hungry owl).

### Method

#### Participants

We tested 90 students (49 women, 40 men, 1 unreported), from the University of Essex aged 18–56 years (*M* = 24.83, *SD* = 6.80), in exchange for £3 payment. The majority of participants were White British (15.7%), White European (23.6%) or Indian (15.7%) followed by Black African (4.5%), Chinese (4.5%), Black Caribbean (1.1%) and Bangladeshi (1.1%). 15.7% of participants did not specify their race. The core design was a 3 (condition: DR, MS, non-existential) X 2 (game order: find the cheese first, escape the owl first) between-subjects Analysis of Covariance (ANCOVA), in which we controlled for baseline levels of enjoyment of computer games (see below). We added the motivation to play each game (find the cheese & escape the owl) as a repeated measures factor (see below). The Ethics Committee of the University of Essex approved the experiment. All the participants provided informed written consent prior to participation.

#### Materials and Methods

Participants completed some filler questionnaires including a demographic sheet that contained a question about how much they generally enjoyed playing computer games (1 = not at all enjoyable, 10 = very enjoyable). We intended for this question to provide a baseline indication of how enjoyable participants found computer games and to control for this variable in subsequent analysis. Next, we randomly assigned participants to the DR or control (MS or dentist) condition. Subsequently, we asked them, in a seemingly unrelated task, to give feedback on two prototype computer games that were under development for future research. They read the descriptions of two games (in counterbalanced order) called ‘find the cheese’ and ‘escape the owl,’ and indicated their motivation for playing each game by responding to the following post-game questions: “How enjoyable do you think this game would be to play?”, “How excited would you be about playing this game?”, and “How much fun do you think this game would be to play?” (1 = not at all, 7 = very much).

In an attempt to encourage participants to differentiate between the two games, we instructed them that: (a) based on their feedback, only one of the mazes would be developed and used in future research, and (b) following their feedback, they would have the opportunity to play the first level of one of the prototype versions of the games. Akin to [64], the notion of advancement (i.e., approaching nourishment) was embedded into the description of ‘find the cheese,’ as participants read that the goal of the game was to navigate a mouse around the maze “in an attempt to seek out a midnight snack of cheese.” In contrast, the notion of security (i.e., avoiding danger) was embedded into the description of ‘escape the owl,’ as participants read that the goal of the game was to navigate a mouse around the maze “to the security of his home” away from a hungry owl.

### Results and Discussion

We averaged the post-game questions into the two types of motivation to play: find the cheese (α = 0.93) and escape the owl (α = 0.88). We conducted a 3 (condition: DR, MS, Dentist) X 2 (game order: find the cheese first, escape the owl first) X (motivation to play: find the cheese, escape the owl) mixed ANCOVA, while controlling for baseline self-reported enjoyment of computer games. Condition and game order were the between-subjects factors, whereas motivation was a repeated measures factor. The main effects for condition, *F*(2, 73) = 2.56, *p* = .09, game order, *F*(3, 73) = 1.40, *p* = .25, and motivation to play the games, *F*(1, 73) = 0.22, *p* = .64, were not significant. The critical Condition x Motivation was significant *F*(2, 73) = 4.85, *p* < .01, η_p_^2^ = 0.12 (Table 3).

**Table 3:** Means and Standard Deviations for Motivation to Play ‘Find the Cheese’ and ‘Escape the Owl’ as a Function of Condition in Experiment 3.

Condition	Find the Cheese Game		Escape the Owl Game	
	Mean	SD	Mean	SD
DR	4.28	1.26	4.26	1.33
MS	4.03	1.64	4.47	1.53
Non-Existential	3.29	1.16	4.31	1.29

To test the interaction between motivation and condition, we conducted simple main effect analyses. The simple main effect of condition for motivation to play “find the cheese” was significant, *F*(2, 73) = 5.46, *p* < 0.01, η_p_^2^ = 0.13. Pairwise comparisons revealed that DR participants were significantly more motivated to play this game compared to participants in the MS condition (*p* < 0.01) and non-existential control condition (*p* < 0.01). In contrast, the MS and non-existential conditions did not differ from one another (*p* = 0.86). The simple main effect for the “escape the owl” game was not significant, *F*(2, 73) = 0.55, *p* = 0.58. In addition, the simple main effect of motivation at each level of condition revealed that DR participants’ motivation to play the two games did not differ *F*(1, 73) = 0.28, *p* = 0.60, whereas both the MS condition *F*(1, 73) = 6.34, *p* < 0.01, η_p_^2^ = 0.08 and non-existential control condition *F*(1, 73) = 17.34, *p* < 0.01, η_p_^2^ = 0.19 were more motivated to play the “escape the owl” game. To determine whether the two control conditions differed in their reasons for their preference for the “find the owl” game, we conducted supplementary and exploratory analyses. We created a relative measure of motivation by subtracting the find the cheese motivation scores from the escape the owl motivation scores for each participant. Positive values reflect greater levels of motivation to play the security-based game, whereas negative values reflect greater levels of motivation to play the nourishment-based game. The bivariate correlation between this new relative motivation measure and the participants’ self-reported baseline level of enjoying computer games was significant, *r*(86) = .26, *p* < .05: the more participants identified themselves as gamers, the more motivated they were to play escape the owl relative to find the cheese. We proceeded to calculating this same correlation within the MS and control conditions separately. The strength of the correlations increased in the control condition, *r*(30) = .64, *p* < .01, and was no longer significant in the MS condition, *r*(28) = -.05, *p* = .80. The two correlation coefficients were significantly different from each other, *z* = 2.91, *p* < .01 (two-tailed).

In summary, participants who contemplated their death in a specific and individuated manner manifested an outcome indicative of identity integration compared to participants in both control conditions. DR participants were equally motivated to play the two maze games, which activated conflicting needs for growth or security. In contrast, participants in the two control conditions expressed greater motivation to play the game that activated security needs, although this preference appeared to occur for different reasons. The more participants in the non-existential condition enjoyed playing computer games generally, the more motivated they were to play the security-based maze, relative to the other maze. This was not the case in the MS condition, suggesting that game preference was not determined by general enjoyment of computer games. Given that the MS manipulation is thought to activate needs, motives, and behaviors that maintain psychological security [66], it is possible that MS participants’ preference was driven by the activation of security-related needs.

## General Discussion

In three experiments, we investigated which existential concerns are likely to facilitate identity integration. As hypothesized, participants who considered their mortality in a specific and individuated manner (via the DR manipulation) consistently demonstrated outcomes indicative of identity integration compared to two control conditions. In Experiment 1, DR participants were equally accepting of the role that positive and negative experiences had played in shaping their current identity. In contrast, control participants devalued these experiences, assigning greater importance to the role of positive than negative life experiences. In Experiment 2, DR participants rated self-serving and other serving values as equally important guiding life principles, whereas MS participants rated the other-serving values as more important. The non-existential control participants demonstrated a descriptive trend that approached significance to prefer self-serving values compared to other-serving values. In Experiment 3, DR participants were equally motivated to play two maze games that activated conflicted needs (growth and security), whereas participants in the control conditions expressed a motivation to play the security maze. Collectively, the findings from these three experiments indicate that specific and individuated consideration of mortality motivates individuals to integrate conflicting aspects into a coherent self-concept.

### Theoretical Implications

Consistent with recent research that addressed the conditions under which mortality awareness facilitates engagement in positive and adaptive behavior [27, 28], the findings suggest that existential contemplation has implications for well-being enhancement. Identity integration is associated with higher levels of well-being [21, 18], whereas a fragmented self-concept is associated with depression and lower self-esteem [23, 24]. It is plausible that specific and individuated mortality awareness would increase a sense of well-being, at least immediately following the mortality reminder.

The DR and MS mortality manipulations had distinct effects on outcomes indicative of identity integration, thus providing further support for the notion that reaction to a mortality reminder may differ depending on the manner in which death is construed [39, 31]. Past research has also found differences between the DR and MS conditions on measures of greed, spirituality, and intentions to give blood [38, 36]. Further investigations will need to focus on the psychological mechanisms responsible for these differences. We know that the MS manipulation also facilitates positive outcomes in certain situations [27], but the outcomes (whether positive or negative) are still considered to function as to reduce death awareness. It remains unclear whether the same psychological mechanisms are responsible for inducing positive outcomes in the DR and MS conditions. It is possible that DR effects are driven by the last two questions of the manipulation [36] that were modeled specifically on the life review process of the near-death experience literature [6]. The life review is considered integral in inducing the positive after-affects and the remedial process that enables persons to approach themselves, others, and the world with a new sense of clarity. Given that these two questions are unique to the DR manipulation, the life review component may be what distinguishes responses to DR from responses to the MS manipulation.

It is also possible that DR elevates self-awareness and that this is a key mechanism underlying the differences. Reading the scenario and answering the questions that follow DR (especially the last two) may render participants more aware of their values and goals in life. A person certainly needs to be self-aware in order to be able to accommodate negative life experiences (Experiment 1), and similarly so when weighing the importance of conflicting values and needs (Experiments 2–3). The MS manipulation motivates people to avoid engaging in activities that induce self-awareness or self-expression [67, 68]. Thus, the differential effects on identity integration outcomes that we obtained may have resulted from increased self-awareness in the DR condition and decreased self-awareness in the MS condition.

### Limitations and Future Research

All three experiments we described relied upon convenience samples of students and university staff, who volunteered to participate. We cannot exclude the possibility that specific and individuated mortality awareness would prime outcomes indicative of identity integration in samples more representative of the general public. For example, it is possible that demographic factors in our sample, such as SES and education level, might constrain the generalizability of our findings. However, we would note that these experiments did include a range of participants from different racial backgrounds, particularly Experiment 2 in which 50% of the sample were Arab and not UK citizens. Furthermore, we would also point to published literature [38] using the DR manipulation that recruited a sample from the general public with a range of participant ages (17–76 years), and still found the hypothesized DR effects.

Although our findings are consistent with the identity integration literature [14, 11, 17], we cannot rule out alternative explanations. For example, it is possible that the DR manipulation increased cognitive conflict between differing beliefs, values, and needs; and thereby the results do not reflect an integrated and holistic self, but rather an increased sense of apathy. We would however, argue that this alternative is unlikely for several reasons. First, Experiment 1 used a validated measure of identity integration [14], and we observed effects similar to those obtained with a manipulation that activated self-directive and autonomous behavior. Second, returning to the initial validation of the DR manipulation, [36] conducted a thematic analysis of the participants’ responses and compared these themes across DR and MS manipulations. Participants did not differ in positive or negative affect, and MS participants wrote more about fearing a painful death even though DR participants had imagined dying in a fire. Furthermore, and in support of the identity integration hypothesis, DR participants engaged in more life reflection and expressed more regrets compared to MS participants. These findings complement the identity integration hypothesis, as DR participants did not demonstrate defensiveness and instead evaluated all facets of their identity (even those they regretted), suggesting that they would be motivated to reconcile differing identity aspects. Third, the published literature on DR [36, 38] also indicates that DR participants are pro-social and other-oriented in their behavior, which again is inconsistent with the notion of a conflicted or apathetic individual. However, it is important that future research examine the mechanisms of this process. [14] observed that self-honesty (or a lack of defensiveness) mediated the relationship between high autonomy and identity integration. The conclusion that DR facilitates identity integration would be strengthened if future studies were also to find that DR reduced defensiveness and in turn predicted identity integration. Similarly, as we discussed earlier, it is possible that the DR manipulation elevates self-awareness relative to our control conditions; therefore it is highly plausible that identity integration would be facilitated through mechanisms that enable unbiased processing of self-relevant information, such as an increase in openness to experience and a reduction in defensiveness.

Our discussion has focused mostly on the psychological benefits of identity integration [21, 17]. However, given that the self-concept is not a monolithic and unitary entity, but is continually reconstructed to fit the demands of the social environment [69], the complexity of the self-concept may have a beneficial role in protecting against the experience of stress and depression [70, 71]. This latter theoretical approach is concerned with how individuals cognitively represent and structure their self-concept, and with the degree of overlap or distinctiveness between these representations. For example, a woman might describe herself as a mother, researcher, and social activist, but perceive these aspects of her identity as relatively unrelated. Put simply, individuals who are high in self-complexity view their identity as consisting of many social roles that are relatively distinct from one another. Those high in self-complexity are less susceptible to stress, depressive symptoms, and physical illness [71], whereas those low in self-complexity are more likely to experience loneliness and dissociative thoughts [72], in the aftermath of a stressful event.

A first glance at this literature may seem to undermine the notion that an integrative and coherent identity facilitates psychological well-being [20, 11]. However, the evidence is far from conclusive. Some researchers have found that self-complexity is negatively associated with indicators of well-being or unrelated altogether [73, 74]. A meta-analysis on the self-complexity literature has produced little support for the stress-buffer hypothesis, instead finding that self-complexity was negatively related to well-being, albeit weakly [75]. Literature reviews have shown that measures of identity integration and self-complexity are empirically distinct, and that self-complexity measures may increase the flexibility with which people describe themselves, whereas identity integration measures may focus attention on behavioral consistency across multiple social roles [76]. In the current work, we relied on the latter theoretical approach, because we were interested in measuring outcomes indicative of identity integration as a function of mortality awareness.

Future research might draw upon recent theoretical models to address how an integrated self-concept is cognitively represented. [76] notes that assimilation (or integration) accounts of the self-concept imply that priming temporarily “moves” or alters the organization of the self-concept. Alternatively, [77] multiple self-aspects framework posits that social identities are assimilated into an associative memory network. Here, integration occurs due to activation of context-dependent personal attributes that are relevant to multiple identities.

## Concluding Remarks

Considering mortality in a specific and individuated manner encouraged participants to acknowledge all facets of their identity—the good, the bad, and the shameful. As a result, they became motivated to integrate conflicting aspects of their identity into a unified and coherent self-concept. The integrative process can result in increased well-being, including a greater sense of continuity, meaning, purpose, and satisfaction with life. The current findings, then, suggest that engaging with the existential reality of death may sometimes serve as a catalyst for well-being.

## Supporting Information

S1 Data Experiment 1: Life Event Integration. (SAV)

S2 Data Experiment 2: Value Integration. (SAV)

S3 Data Experiment 3: Need Integration. (SAV)
